# Revisiting Additional Outcomes in Food Waste Studies: Evidence from Low-Income Households in Chile

**DOI:** 10.3390/nu17142355

**Published:** 2025-07-18

**Authors:** María Isabel Sactic, Andres Silva

**Affiliations:** Escuela de Nutrición y Dietética, Facultad de Ciencias de la Rehabilitación y Calidad de Vida, Universidad San Sebastián, Santiago 7510157, Chile

**Keywords:** food waste, food insecurity, fruit and vegetable purchases, household behavior, randomized experiment, ODS2

## Abstract

Background/Objective: Previous research has measured the impact of interventions on food purchases and food waste separately. Moreover, food waste studies have rarely included food insecurity measurements, which could help develop more comprehensive interventions. The aim of this article is to evaluate the effect of educational videos on food and fruit and vegetable purchases, waste and food insecurity in low-income households. Methods: This study uses an experimental design involving low-income households in Chile to evaluate the effects of three educational videos: videos of (T0) recipes using regular fruits, (T1) refrigerator cleaning instructions, and (T2) recipes using overripe fruits. Results: The videos featuring fruit-based recipes (T0 and T2) increased fruit purchases and reduced fruit waste. In contrast, vegetable purchases and waste increased, especially under the recipe-based interventions. All interventions led to a decrease in food insecurity. Conclusions: An intervention that leads to a reduction in fruit waste can also hide an increase on vegetable waste, as well as changes on purchases and a decrease of prevalence of food insecurity. These findings highlight the importance of measuring fruit and vegetable purchases and food insecurity as complementary outcomes in food waste studies.

## 1. Introduction

Food waste represents one of the most pressing sustainability challenges of our time, with approximately one-third of global food production estimated to be wasted annually [1]. Previous research indicates that food waste occurs at various stages of the production chain, with variations depending on the country’s level of development [2]. In developing nations, the majority of waste occurs early in the production process, largely due to limited access to technology and resources for effective food management [3]. In contrast, developed countries experience higher rates of food waste at the household level, often attributable to consumer behavior [4].

Reducing food waste faces significant challenges due to substantial gaps in the information available on the topic [5]. To address this issue, the FAO created the Food Loss Index, primarily focusing on the losses occurring during production and supply before reaching the end of the chain [6]. In contrast, there is little comparable information on the waste generated at the household level. This is mainly due to the great diversity of the methods used to estimate waste, in addition to the fact that there is little consensus on the definition of food waste, and therefore measurements vary [7,8]. Furthermore, information is even more limited in developing countries due to the lack of applied research and primary-data-based studies [9].

According to [10], global food waste averages at 65 kg per capita annually, with differences observed across income levels, ranging from 43 kg in low-income countries to 163 kg in high-income countries. Some nations, such as New Zealand, the United States, and Australia, report even higher figures, with up to 183 kg of waste per capita per year. Moreover, variations exist in the types of food wasted within households. Ref. [11] found that in the European Union, the most commonly wasted food categories include fruits, vegetables, and cereals. Similarly, ref. [12] reported that in a study encompassing two Swedish cities, fruits and vegetables accounted for up to 30% of household food waste. In a study conducted in a Latin American country, ref. [13] found that among a sample of Uruguayan households, 45.9% of the respondents reported discarding prepared foods as their most recent waste event. Additionally, 14.6% reported discarding vegetables, while 8.7% reported discarding fruits.

Reducing food waste can contribute to feeding the population, ensuring the livelihoods of households along the agro-food supply chain, and accomplishing environmental goals [14]. In this sense, food waste can be thought of as a byproduct of a more complex system. If an intervention is likely to impact more than an outcome, then we argue that we need additional measurements. The aim of this article is to evaluate the effect of educational videos on food and vegetable (FV) purchases, waste and food insecurity in low-income households. For the first time, we are able to assess the impact of education on FV purchases, FV waste, and food insecurity. We argue that these three outcomes are complementary since from a public policy point of view, we would like not only to reduce FV waste but we would also like to reduce FV waste while increasing FV purchases and decreasing food insecurity. Moreover, for the first time, we can assess the impact on fruit and vegetables separately. We conducted an experimental study that included a control group that watched a video of regular FV cooking recipes (T0), a group exposed to a video with refrigerator cleaning instructions (T1), and a group that watched a video on recipes on cooking with overripe FVs (T2).

Studies have shown that the amount of food wasted in households is significantly correlated with factors such as household income, household size, and age [15]. In addition to sociodemographic determinants, previous research has also reported behavioral determinants, food skills, and awareness to explain household food waste [15,16,17]. Also, psychological determinants, such as mood and stress, and attitudes and beliefs can play an important role in food waste [18].

Household income has been pointed out as having a relevant role in food waste. Prior research indicates that high-income households tend to generate more food waste than their lower-income counterparts [19]. High-income households tend to consume more fresh foods and allocate a smaller proportion of their income to food expenses. Fresh foods, compared to processed options, generally contribute to higher levels of food waste [20]. In this context, fresh FVs tend to comprise a larger proportion of food waste. Additionally, due to its positive income elasticity, food waste can be classified as a normal good: as income rises, the quantity of food waste increases [21].

Household income also plays a significant role in FV purchasing patterns, with certain FVs following a socioeconomic gradient. For example, whole fruit consumption is often associated with higher levels of education and income, while the intake of 100% fruit juice is more common among lower socioeconomic groups. Similarly, white potato consumption tends to be linked with a lower income, whereas the consumption of leafy salad greens is notably higher among higher-income households [18]. Furthermore, even though few individuals meet the recommended “five-a-day” FV intake, some people report satisfaction with their current FV consumption levels [22]. This may indicate a need for greater education to help individuals recognize the importance of incorporating more FVs into their diets.

Previous research had also consistently found that more educated households purchase and consume more FVs: the study [22], using data from four Latin American countries, found that satisfied respondents consumed significantly more FVs than unsatisfied ones. A satisfied respondent was considered as someone who expressed being satisfied with their current FV consumption level. The authors found that 70.1% of the respondents were satisfied with their current levels of FV consumption, while only 43.3% achieved the five-a-day recommendation. In this sense, desirable FV consumption in most cases was lower than the public health recommendation, while education led to a significant effect on both desirable and current FV consumption levels.

## 2. Materials and Methods

Households were recruited through outreach promoted by local municipal authorities and social media channels, and interested participants registered voluntarily. The data collection then proceeded in three main phases throughout 2022 and 2023. The first phase involved developing online questionnaires, which were distributed through social media channels to test and validate questions related to food purchases, particularly FV purchases, and socioeconomic data. This phase provided insights for refining and validating the questions to include in the final study.

The second phase consisted of the first pilot implementation of the experiment in households. The pilot was conducted in 33 households in Talagante, a municipality in the Metropolitan Region of Santiago, Chile. Each household was asked to complete an initial survey regarding its food procurement habits and sociodemographics. Then, the households kept record of their FV purchases and FV waste for seven consecutive days. Subsequently, the respondents were randomly assigned into treatment groups, and videos were sent as interventions. Respondents were then asked to repeat the seven-day tracking of their FV purchases and food waste. Finally, the households were requested to complete an exit survey, which included additional characterization questions and feedback on the data collection process.

Lastly, the third phase involved the final data collection, incorporating the feedback and insights gained from the previous two stages. To facilitate this, this study was promoted through social media and with the support of local municipal authorities to ensure broader participation. Household participation was not restricted by municipality, although greater outreach efforts were made in peripheral areas and several middle- and low-income municipalities within the Santiago Metropolitan Region. The final sampling took place during November and December of 2023, following the same steps as those in the pilot.

**Figure 1:**
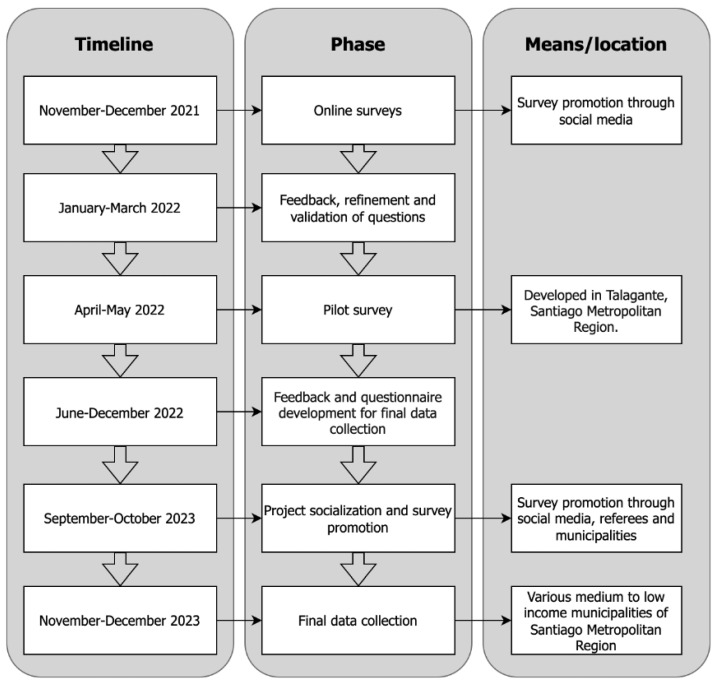
Data collection process flowchart.

All registered respondents were over 18 years of age, with an average age of 44.5 years and ages ranging from 23 to 64 years. The sample consisted predominantly of women, who represented 88% of the respondents. No identifying information was requested from the respondents to ensure anonymity and protection of their data. Additionally, the informed consent outlined that respondents would receive an incentive payment in the form of a gift card upon the completion of each stage of the study. Also, the respondents were automatically entered into a draw for a larger gift card upon successful completion of all stages of the study. This follows the standard practice of conducting interventions with real monetary incentives, as they yield more accurate measurements [23]. Figure 1 shows the summary of the data collection process.

### Data

The dataset consists of two main sections. The first section contains a household characterization, including information about the head of household, household composition, and other relevant factors documented in the literature related to FV consumption and waste. These factors include cooking skills, the presence of pets in the household, and other food-related behaviors. The second section comprises weekly records of FV purchases and waste. FVs were recorded in two formats, depending on the respondent’s preference: either by their exact weight or by the quantity and size of each fruit or vegetable.

For entries recorded by weight, all data were standardized to grams. In cases where the respondents recorded the quantities and sizes of FVs, conversion tables were used to convert these entries into weights, ensuring consistency across all records. Grams were chosen as the standard unit to facilitate the calculation of the portions purchased and discarded by each household.

Given the experimental design, the data was collected in multiple stages, resulting in varying numbers of observations at each stage due to respondent attrition. To ensure consistency in the reported data, only the 48 households that completed all stages of the data collection were included in the final dataset. Additionally, for the FV purchase records, weekly values were confirmed with each participant via telephone to ensure the accuracy of the reported information.

## 3. Results

Table 1 shows the descriptive statistics of the sample. Most of the respondents were women and on average 45.1 years old. On average, a household had 3.6 members. According to data from the *Encuesta de Presupuestos Familiares* IX (2021–2022), in the first three quintiles in the metropolitan area, a household had 2.7 members on average. In terms of household income, 68% of the respondents earned less CLP 1.0 million, which was equivalent to USD 1100–1200 in December 2024. Therefore, four out of five respondents in the sample earned a little more than a thousand dollars a month.

Table 1 also shows that these households consume vegetables more often than fruits. Previous research has found that low-income households purchase more vegetables than fruits. We were not able to assess the causal determinants that explained this fact. It could be that low-income households cook more, and more often, than high-income households. The latter also rely more on food-away-from-home alternatives. Moreover, fruits tend to be more expensive than vegetables.

Table 2 shows the FV purchases by treatment group. In T0, the respondents watched a video with recipes using regular fruits. In T1, the respondents watched a video with refrigerator cleaning instructions. In T2, the respondents watched a video of recipes using overripe fruits. W1 and W2 refer to week 1 and 2, respectively. T0 and T2 led to a larger increase in fruit purchases than vegetable purchases, which may be explained by the fact that these videos showed fruit-based recipes. However, T1 led to a decrease in fruit purchases. This shows that the video content does play a relevant role in the type of food.

In a similar way, Table 3 shows the FV waste by treatment group. In T0, the respondents watched a video with recipes using regular fruits. In T1, the respondents watched a video with refrigerator cleaning instructions. In T2, the respondents watched a video with recipes using overripe fruits. W1 and W2 refer to week 1 and 2, respectively. All three videos led to a decrease in fruit waste and an increase in vegetable waste. Considering Table 2, these households also increased their vegetable purchases, which shows the relevance of considering both outcomes before making policy recommendations.

We found that the T1 video, with refrigerator cleaning instructions, led to a decrease in fruit purchases and also fruit waste and a relatively small increase in vegetable purchases and then vegetable waste. In other words, cleaning instructions do not directly impact how food is prepared. Differently, we found that the T0 and T2 videos (videos with recipes) were able to increase fruit purchases and decrease fruit waste. In the case of vegetables, we found that the T0 and T2 videos, videos with recipes, were able to increase vegetable purchases and increase vegetable waste. It may be the case that video recipes increase cooking, which leads to food waste.

Therefore, the education videos led to distinctive effects on purchases and waste for fruit and vegetables separately. In general, fruit-based recipes increased fruit purchases and decreased fruit waste. In addition, videos on refrigerator cleaning instructions led to a decrease in FVs and then a decrease in FV waste. Our results show that educational videos can be an effective way to change behavior, while the content would lead to different effects on the type of food.

Finally, Table 4 shows the prevalence of food insecurity for each treatment. The results show that both types of educational videos, of cooking recipes and refrigerator cleaning instructions, lead to a decrease in food insecurity. Therefore, depending on the content, educational videos have an effect on food purchases and food waste, and in all cases, education videos help to reduce food insecurity. However, food waste and food insecurity do not have a linear relation. As we have presented, we found that fruit waste can be reduced while food insecurity decreases. However, we also found that vegetable waste can be increased while food insecurity decreases.

## 4. Discussion

Despite FVs’ relevance as part of a healthy diet, most previous research has analyzed FV waste as a single type of food, while they are different, at least in terms of their nutritional composition, preparation requirements, and health benefits, as presented by [24]. Moreover, the selection of ripe fruits may require some expertise, while vegetable may be easier to pick at the point of purchase. Therefore, in most cases, FVs have relevant differences which are overlooked in making policy recommendations.

To the best of our knowledge, this is the first study to simultaneously assess the effect of education on FV purchases, FV waste, and food insecurity, providing a more comprehensive understanding of the FV procurement process. From a public policy perspective, an intervention that reduces FV waste but also decreases FV purchases may not be desirable. Our results show that the educational videos increased fruit purchases, decreased fruit waste, and increased vegetable purchases, although vegetable waste also increased. This pattern may reflect the greater focus on fruits in the educational videos, particularly in T0 and T2, which included fruit-based recipes and could have led to stronger effects on fruits compared to those on vegetables. However, our database does not allow us to argue causality. In all cases, we found a reduction in food insecurity. However, as this analysis is exploratory and the dataset’s size does not allow us to assess the link between education, food waste, and food insecurity in depth, the underlying mechanism in place still remains an area for future research.

Table 2 shows different effects for FV purchases, which were expected to some extent. Typically, FVs are commonly aggregated as a single type of food, but they differ in their nutritional content and in the skills and effort required to consume them. Previous research in Chile has reported that fruits that require minimal preparation are more widely consumed [25]. Moreover, external characteristics such as freshness, color, and glossiness influence fruit purchase decisions [26]. Additionally, ref. [27] showed that most fruits are eaten raw, while vegetables are less frequently consumed raw, implying that vegetables generally require more preparation. For example, in their study, around 99% of fruits were eaten raw, compared to only 61% of vegetables. Moreover, ref. [28] found that foods requiring longer preparation times may be consumed less frequently, as higher time demands for food preparation are associated with healthier dietary patterns but can act as a barrier when time resources are limited. In summary, while our findings describe differences in the effects of the educational videos on FV purchases, it is important to note that this study is descriptive. Other unmeasured factors, such as prior knowledge, household characteristics, cultural habits, food packaging, and individual motivation, may also have influenced purchasing behaviors. Future research could explore these aspects in more detail to understand the mechanisms behind food purchasing and waste reduction decisions betters.

Our study focused on an educational video intervention, highlighting its potential impact on food purchases, waste, and food insecurity. However, it is important to note the limitations of our study. The sample size was small and based on voluntary participation, resulting in self-selection bias, with most households coming from the same commune in Santiago. Additionally, we did not collect information on the education levels of the participants, which limited our ability to analyze whether educational attainment influenced the interventions’ effectiveness. Moreover, while our intervention was centered on educational content, other approaches such as behavioral nudges have also shown potential to reduce household food waste. For example, ref. [29] found that interventions using nudges, such as providing feedback on waste amounts, promoting meal planning, and encouraging social comparisons and challenges, can effectively support households in reducing food waste by facilitating behavior changes in daily food practices.

## 5. Conclusions

Using an experimental approach, we assessed the effect of educational videos on FV purchases, FV waste, and food insecurity. Our educational videos showed simple recipes using fresh fruits (T0 and T2) and refrigerator cleaning instructions (T1). We found that the video recipes led to an increase in fruit purchases and a decrease in fruit waste. However, the educational videos led to an increase, although smaller compared to that for fruits, in vegetable purchases and also an increase in vegetable waste, which could be associated with the increase in vegetable purchases. In all cases, the educational videos led to a decrease in food insecurity.

Our results show that households react after watching video recipes, with changes not only in FV waste but also in FV purchases and food insecurity. These findings suggest that in studies like ours, including additional outcomes such as food purchases and, when relevant, food insecurity can provide deeper insights into the multifactorial nature of food waste. Equally, we recommend that future experimental studies analyze fruits and vegetables separately, as their effects may differ. These exploratory data suggest that there are differences in food preparation practices between food-secure and food-insecure households. Previous evidence indicates that foods that require longer preparation times tend to be consumed less frequently, which may contribute to disparities in dietary intake. Future research should examine these associations in greater depth.

## Figures and Tables

**Table 1:** The descriptive statistics of the sample.

Variable	Mean	SE
**Respondent characteristics**		
Gender (0 = man, 1 = woman)	0.879	(0.323)
Age (years)	44.515	(9.236)
**Household characteristics**		
Household size	3.636	(1.421)
Households with children	0.636	(0.485)
Households with pets	0.756	(0.432)
**Household income**		
Less than CLP 500,000	0.343	
CLP 500,000–1,000,000	0.469	
CLP 1,000,000–2,000,000	0.156	
CLP 2,000,000–3,000,000	0.031	
**Fruit consumption**		
1–2 days a week	0.059	
3–4 days a week	0.500	
More than 5 days a week	0.441	
**Vegetable consumption**		
1–2 days a week	0.059	
3–4 days a week	0.441	
More than 5 days a week	0.500	
**Food insecurity prevalence**		
Moderate week 1	20.973	(34.249)
Moderate week 2	14.413	(25.429)
Observations	48	

Note: Values represent means and standard errors (SEs) for continuous variables.

**Table 2:** Fruit and vegetable purchases by treatment group.

Treatment	Total			Fruit			Vegetables		
	**W1**	**W2**	**Diff**	**W1**	**W2**	**Diff**	**W1**	**W2**	**Diff**
T0	10.882	15.465	4.586	5.465	8.309	2.844	5.417	7.156	1.739
T1	12.484	12.213	−0.271	8.333	6.665	−1.668	6.226	6.658	0.432
T2	11.352	15.493	4.141	6.826	8.654	1.828	5.208	6.838	1.630

*Note*: Values represent the average number of portions purchased per day per household. T0: regular fruit recipe video group; T1: refrigerator cleaning video group; T2: overripe fruit recipe video group. W1 and W2 refer to week 1 and week 2, respectively.

**Table 3:** Fruit and vegetable waste by treatment group.

Treatment	Total			Fruit			Vegetables		
	**W1**	**W2**	**Diff**	**W1**	**W2**	**Diff**	**W1**	**W2**	**Diff**
T0	0.559	0.839	0.338	0.545	0.509	−0.036	0.331	0.595	0.264
T1	1.230	1.448	0.218	0.872	0.790	−0.082	0.895	1.237	0.342
T2	1.257	1.827	0.570	0.968	0.952	−0.016	0.482	1.618	1.136

*Note*: Values represent the average number of portions wasted per day per household. T0: regular fruit recipe video group; T1: refrigerator cleaning video group; T2: overripe fruit recipe video group. W1 and W2 refer to week 1 and week 2, respectively.

**Table 4:** Probability of food insecurity by treatment group.

Treatment	Week 1	Week 2	Diff
T0	23.405	19.229	4.176
T1	15.849	12.993	2.856
T2	13.515	10.490	3.025

*Note*: Food insecurity prevalence is estimated using the FIES questionnaire developed by FAO.

## Data Availability

The data is available from the corresponding author upon request.

## Abbreviations

The following abbreviations are used in this manuscript:

FV	Fruits and vegetables
T0	Treatment 0, control group
T1	Treatment 1, refrigerator cleaning instructions
T2	Treatment 2, overripe FV cooking recipes
